# The Role of Bridging Therapy in Hepatocellular Carcinoma

**DOI:** 10.1155/2013/419302

**Published:** 2013-12-19

**Authors:** Roberto Galuppo, Angie McCall, Roberto Gedaly

**Affiliations:** Department of Surgery, College of Medicine, University of Kentucky, Lexington, KY 40536-0293, USA

## Abstract

Hepatocellular carcinoma (HCC) is the most common primary malignancy of the liver accounting for 7% of all cancers worldwide. Most cases of HCC develop within an established background of chronic liver disease. For that reason, liver resection is only possible in selected patients. Liver transplantation has become the treatment of choice in patients with HCC, end-stage liver disease, and significant portal hypertension. Shortage of organ donors has resulted in overall increase of waiting list time with increased risk of dropout due to tumor progression. Neoadjuvant therapies have emerged as an alternative to control tumor growth in patients while waiting. The aim of this study is to review the literature on the role of bridging therapy and downstaging prior to liver transplantation in patients with HCC. We are also presenting our single-center experience of 96 patients undergoing transplantation for HCC with and without bridging therapy.

## 1. Introduction

Hepatocellular carcinoma (HCC) is the most common primary malignancy of the liver, the sixth most common cancer (749,000 new cases each year), and the third cause of cancer-related death worldwide [1].

In the western world, most cases of HCC develop within an established background of chronic liver disease and portal hypertension (70%–90% of all patients). Liver resection is only possible in selected cases due to the high incidence of morbidity and mortality in patients with cirrhosis and elevated portal pressures. Liver transplantation (LT) has become the treatment of choice for patients with HCC and end-stage liver disease, as it has the advantage of eradicating the tumor and the premalignant cirrhotic liver. Recurrence after LT ranges from 8% to 15% when a specific criterion for selection of patients is used. Surgical resection and ablation therapies have been associated with much higher rates of recurrence [2].

After Milan criteria were established (single nodule less than 5 cm or 3 nodules less than 3 cm), excellent results have been reported with survival in the range of 60%–70% at 5 years [3, 4]. Nonetheless, shortage of organ donors is increasing the waiting time and consequently leading to 30%–40% dropout per year because of tumor progression [5].

Therefore, the practice of treating HCC patients with locoregional therapies before LT, as they are waiting to be transplanted, has become standard in most centers [6].

We reviewed the literature on the use of locoregional therapies prior to liver transplantation and analyzed patients undergoing transplantation for HCC in our institution with emphasis on bridging therapy.

## 2. Locoregional Therapies as a Bridge to Liver Transplantation

Locoregional therapies play a major role in the current therapeutic management of HCC. They encompass a broad range of modalities including radiofrequency ablation (RFA), percutaneous ethanol injection (PEI), transarterial chemoembolization (TACE), liver resection, and microwave ablation [7, 8].

The most significant problem in patients with HCC on the waiting list is the possibility of tumor progression. For this reason, most centers started to use locoregional or neoadjuvant therapies to control tumor growth in patients while waiting. Although bridging therapies using ablation, TACE, resection, or combination treatments have been used by different transplant centers worldwide, the real impact and indication of any type of neoadjuvant treatments are still in debate. Some authors propose that patients with HCC waiting for more than 3 to 6 months should be treated [9, 10]. Various studies have suggested that treatment of HCC prior to LT in patients with a waiting time less than 6 months is not associated with an impact in patient survival or tumor recurrence and raises the question of cost effectiveness of treatment [11]. The overall risk of dropout in patients with diagnosis of HCC waiting for liver transplantation has been reported in the range of 15% to 30% at one year. New studies have reported that a lower incidence of dropout in the range of 0% to 25% may be related to the use of neoadjuvant therapies.

However, locoregional or neoadjuvant treatments prior to liver transplantation have been used to reduce tumor burden if patients are considered to be outside criteria for transplantation in a strategy called downstaging. The group from Paris, France, at L'Hopital Paul Brousse, initially recommended this strategy in 1997. They observed higher rates of survival in TACE responders than in nonresponders in an analysis of patients with more than three nodules or nodules greater than 3 cm [12]. LT was then performed only in patients that fulfilled Milan criteria after treatment. Furthermore, several prospective studies have reported good patient survival compared to patients undergoing LT without prior intervention.

### 2.1. Ablation

The use of radiofrequency ablation (RFA) for the treatment of liver tumors started in the early 1990s both in Europe and in the USA [13]. Radiofrequency ablation (RFA) is a form of locoregional therapy that utilizes a high-frequency alternating current using a probe inserted into the tumor.

The radiofrequency waves are converted into thermal energy within the conducting tissue, destroying the tumor [14]. Early experiences reported high risk of seeding, making RFA not an appealing treatment in patients while waiting to be transplanted. However, in the last few years, a well-conducted cohort study demonstrated that seeding is a rare event [7].

Percutaneous ethanol injection was used for several years in Asia and then in Europe and the USA to treat HCC with excellent results in patients with small tumors and was usually limited to less than 3-4 lesions. PEI was used for many years as treatment and more recently as bridging therapy in patients waiting for liver transplantation.

Other authors previously published that PEI can produce similar amounts of tumor necrosis although it requires a significant additional number of sessions compared to RFA. For that reason, most centers in the USA use RFA as the preferred method of ablative therapy to treat or as neoadjuvant treatment in patients with HCC. Llovet et al. demonstrated in a Markov model that ablation in patients awaiting liver transplantation had a possible gain in life expectancy and cost per year of life saved.

Several studies [7, 15–19] validated the efficacy of RFA as the most promising therapy for bridging patients to transplantation. Patients with single nodules treated with RFA showed drop-out rates ranging from 0% to 21%, while historical nontreated controls showed drop-out rates at 1 year of 30% [20].

RFA has few theoretical advantages over TACE. It has less posttreatment discomfort and simple percutaneous access, and it can be applied in patients with mild-moderate to severe liver dysfunction [21].

Ethanol injection could still be recommended in cases where radiofrequency ablation is not technically feasible such as lesions located close to large vascular structures where heat sinking effect can be observed (around 10%–15%) [22].

Other therapies such as microwave ablation have been used in some centers, but they are still under investigation. Irreversible electroporation is another ablation technique that has been used in patients with tumors abutting major vascular structures where heat sink and collateral damage must be avoided. The role of this technique as neoadjuvant treatment in HCC patients awaiting transplantation is still to be determined [23].

### 2.2. Transarterial Chemoembolization

TACE combines two different therapeutic approaches. First, application of chemotherapeutic agents is usually mixed with lipiodol as a vehicle into the feeding vessels of the tumor. Lipiodol is an oily contrast used for lymphographic studies and is selectively retained within the tumor, raising the exposure of neoplastic cells to chemotherapy. Second, the feeding artery is occluded by microparticles inducing ischemia and a prolonged exposure to the chemotherapeutic agent. Hepatic artery obstruction is usually achieved by Gelfoam particles, but not polyvinyl alcohol (PVA), starch microspheres, metallic coils, and autologous blood clots [24]. Drug-eluting beads (DEB) are a novel system consisting of PVA beads (500–700 *μ*m) that are specifically designed to release chemotherapy at a slow rate.

Survival of patients with advanced HCC not suitable for radical therapies treated with TACE is improved compared with best supportive care [25]. Side effects range from the postembolization syndrome up to hepatic insufficiency, which is very rare. The main purpose of TACE as a bridge to transplantation is to reach local tumor control until a donor organ becomes available [26].

TACE is the preferred single-treatment modality in downstaging protocols, especially for multifocal tumors [10]. But combined modalities of TACE, RFA, PEI, and resection seem to downstage patients more effectively than TACE alone [27].

TACE is the most commonly used form of neoadjuvant therapy, alone or in combination with ablation/resection, in patients listed for LT or included in a program of downstaging [6].

The use of external beam radiation therapy in HCC treatment has been limited by the low radiation tolerance of the nontumoral cirrhotic liver. Transarterial radioembolization (TARE) instead has been recently used in the management of HCC not suitable for curative treatment with similar indications as those of TACE. Radioembolization consists of infusion of radioactive substances including microspheres containing yttrium-90 (Y90), iodine-131-iodized oil, or similar agents into the hepatic artery [9]. It represents an interesting alternative, as it appears to induce a more efficient decrease in tumor size, with a shorter time to response (4.2 versus 10.9 months) [28]. In addition, TARE could be performed in cases of portal vein thrombosis, a relative contraindication to TACE [29].

There are no randomized control trials available to assess the real place of this treatment modality in HCC patients or as a bridge to transplantation.

### 2.3. Resection

Surgery is the mainstay of HCC treatment. Resection and transplantation achieve the best overall outcomes in well-selected candidates (5-year survival of 60%–80%) and compete as the first options in patients with early tumors.

Resection is the first-line treatment option for patients with solitary tumors and very well-preserved liver function, defined as normal bilirubin with either hepatic venous pressure gradient ≤10 mmH or platelet count ≥100,000. While selected patients with cirrhosis are best treated with resection, patients with ESLD and portal hypertension are at increased risk of morbidity and mortality compared to noncirrhotic counterparts. For these reasons, only 20%–30% of patients with cirrhosis, portal hypertension, and HCC are candidates for resection. Selection of the ideal candidate requires an adequate assessment of the liver functional reserve, tumor extension, and risk of postoperative complications and mortality.

Several different systems have been used to try to address risk of morbidity and mortality after resection in patients with portal hypertension and cirrhosis. The Child-Pugh classification system initially permitted assessment of liver function. Nowadays, more sophisticated measurements such as indocyanine green retention rate at 15 min (ICG15) [30] and hepatic venous pressure gradient (HVPG) as direct measurements of relevant portal hypertension are utilized [31, 32]. Low platelet count has been confirmed as a strong independent predictor of mortality in patients with HCC and cirrhosis [22, 33].

Resection can be used as a therapy for HCC prior to LT in different settings. Initially, resection can be used as a first-line treatment for patients with small HCC and preserved liver function. Also resection could help refine the selection process for LT according to detailed pathological examination of the tumor and the surrounding liver parenchyma. It could help in selection of candidates for LT in patients with tumors slightly outside the Milan criteria but with histological features of good prognosis or in denying LT in patients within the Milan criteria but with histological features of poor prognosis such as undetected macrovascular invasion.

Several groups considered surgical resection as one of the bridging treatment modalities prior to LT. Other groups consider liver resection as one of the options for downstaging patients with tumors outside the transplantation criteria [34].

Tumor recurrence represents one of the major difficulties after resection. The pattern of recurrence influences subsequent treatment allocation and outcomes. In these instances, the patients could be reassessed, staged, and retreated accordingly.

## 3. Downstaging

The role of downstaging HCC patients prior to LT is not very clear. There are several centers in the USA and Europe trying to use different types of neoadjuvant therapy to treat HCC to decrease tumor burden to fulfill usually Milan criteria and then perform liver transplantation. The type of therapy that should be used and the upper limit of tumor size that should be downstaged are not clear. Some authors have recommended putting the candidate on hold until downstaging by local ablation and/or chemoembolization is achieved and maintained (disease stability) for a period of at least three months [22]. However, the real waiting time after successful downstaging is not clear.

There is not a single randomized control trial or a large cohort study available on patients consistently treated and properly followed. Various prospective small studies suggested that downstaging tumors to reach Milan criteria using RFA and/or TACE achieves 5-year survival rates similar to those within the conventional criteria after LT.

Ravaioli et al. presented a prospective study providing validation of a downstaging protocol, in which patients, initially excluded by the conventional transplant criteria, undergoing successful downstaging obtained satisfactory survival after LT [35]. Still efficacy of downstaging in patients exceeding conventional criteria is strictly related to tumor size and number at presentation. The bigger the tumor bulk, the lower the efficacy of downstaging in terms of tumor response [10].

Considering the existing information, downstaging of patients beyond Milan criteria still remains controversial, and criteria for selecting candidates are not well established. Chapman et al. considered most patients beyond Milan criteria and did not exclude a priori any patients. This group reported that 17 out of 76 patients were transplanted after successful downstaging. They reported comparable outcomes in these 17 patients to those in patients within Milan criteria [36]. Yao et al. restricted downstaging eligibility criteria to one lesion >5 cm and up to 8 cm; 2 to 3 tumors with at least one lesion >3 cm but not exceeding 5 cm, with total tumor diameter up to 8 cm; or 4 to 5 lesions with none >3 cm with total tumor diameter less than 8 cm. Using these criteria as an upper limit for downstaging, 57.4% (35/61) of their patients were successfully downstaged and transplanted [37]. Ravaioli et al. prospectively analyzed patients with a single HCC 5 cm to 6 cm, 2 lesions ≤5 cm, or less than 6 HCC lesions ≤4 cm and sum diameter ≤12 cm, and they achieved a 67% transplantation rate (32/48 patients). They achieved similar outcomes in patients treated versus those within conventional Milan criteria [35].

## 4. Our Experience

In the Transplant Center at the University of Kentucky, the strategy for management of HCC has evolved with a multimodality algorithm approach similar to that adopted in other centers [38].

We retrospectively analyzed our experience of 96 consecutive patients undergoing transplantation for HCC from September 1999 to September 2011. Of the 96 patients, 78 were males (81.3%). As expected, hepatitis C (HCV) was the most common indication in this group (48 patients, 50%), followed by alcoholic liver disease (40 patients, 41.7%). Among the 96 patients who underwent LT, 31 patients were identified as having incidental HCC on the explanted livers, undetected on preoperative imaging (Table 1).

A total of 49 patients (54.4%) had single lesions. The mean diameter of the greatest lesion was 2.3 cm, ranging from 0.7 to 7.6 cm. Vascular invasion was found in 22 patients (22.9%).

**Figure 1:**
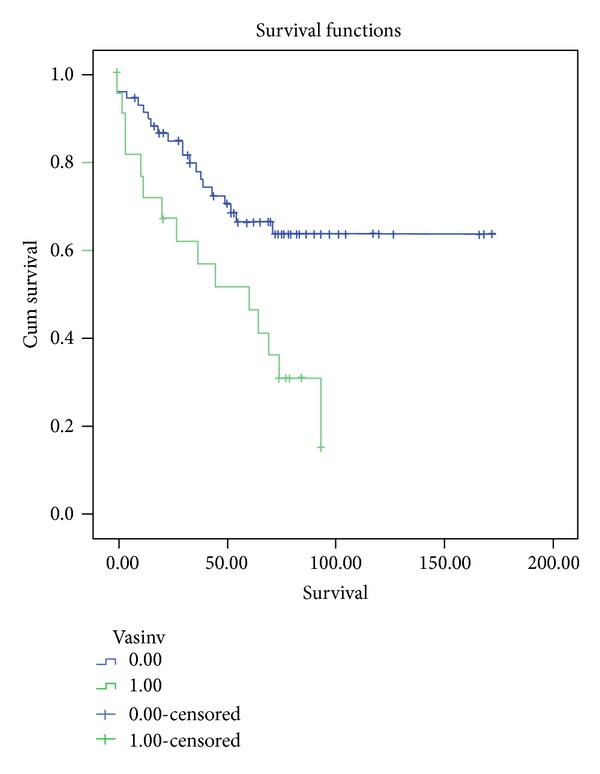
This graphic shows survival among patients undergoing LT with and without vascular invasion. Survival is significantly better in those patients transplanted without vascular invasion (*P* < 0.05).

**Figure 2:**
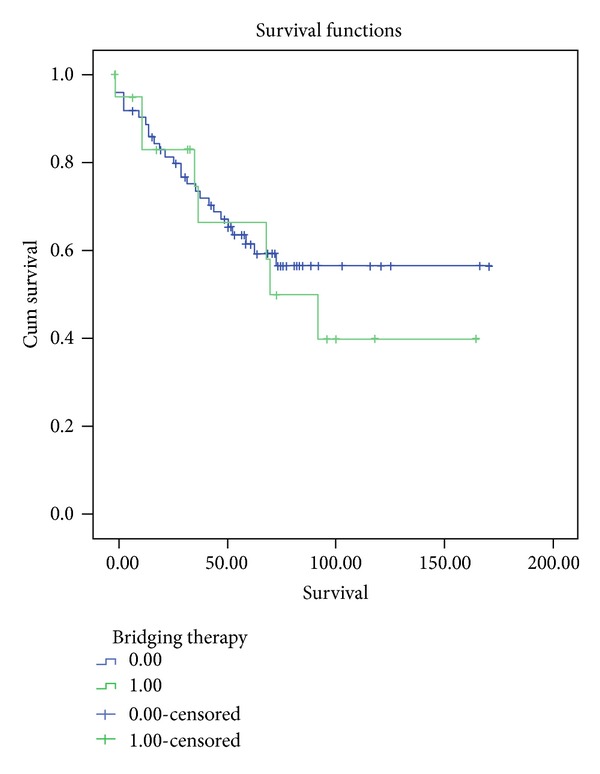
This graphic demonstrates survival among patients undergoing LT with and without bridging treatment. Similar survival rates were observed in patients transplanted with and without BT (*P* = ns).

Analysis of survival was performed using the Kaplan-Meier method. For intention-to-treat survival, all 96 patients were followed up from the time of listing to death or last followup. Overall survival rates of 89.2%, 74.1%, and 62.1% at one, three, and five years were observed, respectively. As expected, patients with vascular invasion had significantly worse survival compared to those patients with tumors without vascular invasion (*P* < 0.005). Rates at one, three, and five years for patients without vascular invasion were 90.9%, 77.6%, and 66.1%, respectively, while for patients with vascular invasion survival was considerably lower; 71.6%, 61.7%, and 46.3% (Figures 1 and 2).

We compared patient characteristics in individuals undergoing bridging therapy with those without preoperative treatment (Table 2). Patients characteristics were similar in both groups other than tumor size greater than 3 cm that was more common in patients in the BT group (*P* < 0.005).

Nineteen liver transplant candidates representing 29.2% of known HCC cases were treated with locoregional therapies (Table 2). Of those patients undergoing neoadjuvant treatments, 10 were treated with RFA (15.4%), 8 with TACE (12.3%), 1 with resection (1.5%), and 1 with RFA/TACE combination (1.5%).

Recurrence was seen in 13 patients representing 13.2% or our cases transplanted for HCC. Survival and recurrence rates were similar in treated patients versus nontreated individuals (*P* = ns).

## 5. Discussion

Liver transplantation represents the best curative option for HCC and cirrhosis as it has the advantage of removing the tumor and treating the underlying disease. Despite that, HCC can progress significantly in patients awaiting LT. It has been reported an approximate doubling time for these cancers of around 6 months [39]. As a consequence, tumors can grow beyond conventional criteria, increasing the possibility of microvascular invasion and occult metastasis and further worsening the patient condition.

Recent advances in imaging techniques, especially with the use of liver-specific contrast MRI, are making diagnosis of HCC and quantification of the amount of disease more accurate. Gadolinium-ethoxybenzyl-diethylenetriamine pentaacetic acid (Gd-EOB-DTPA) is a liver-specific magnetic resonance imaging contrast agent that has up to 50% hepatobiliary excretion in the normal liver. After intravenous injection, Gd-EOB-DTPA distributes into the vascular and extravascular spaces during the arterial, portal venous and late dynamic phases and then progressively into the hepatocytes and bile ducts during the hepatobiliary phase. The role of gadolinium-ethoxybenzyl-diethylenetriamine pentaacetic acid-enhanced magnetic resonance imaging (EOB-MRI) in the management of HCC is still to be determined. However, most centers are now using EOVIST when regular dynamic CT/MRI images do not show conclusive findings.

Selection criteria for liver transplantation in HCC patients are still controversial [22, 40–43]. Although initial LT results in nonselected patients with HCC were discouraging, subsequent series proved that better results could be achieved by employing defined selection criteria [44]. The criteria proposed by Mazzaferro et al. in 1996 are used as a basic stratification tool by numerous transplantation centers worldwide [2]. Using these criteria, a patient with a single tumor measuring 5 cm or less, or three or fewer nodules each smaller than 3 cm, would be a candidate for LT. Excellent 5-year survival rates in the range of 60%–70% or higher have been reported when these criteria are followed. Subsequently, the University of California at San Francisco reported 57% survival for patients with HCC who exceeded Milan criteria but were within the limits of their expanded criteria, including patients with solitary lesions less than 6.5 cm in size or up to three tumors with the largest not more than 4.5 cm and a combined tumor diameter of not more than 8 cm [45]. The TNM criteria by Marsh et al. at the University of Pittsburgh are based on HCC characteristics including microvascular or macrovascular invasion, lobar distribution, tumor size, and lymph node involvement, and they seemed to provide a more clear-cut discrimination with respect to post-OLT survival for each TNM tumor stage (I to IV) [46].

Yao et al. compared the previous three proposed criteria and supported a modest expansion of the tumor size limits of the Milan criteria (the UCSF criteria) while still preserving acceptable survival after OLT. The expanded criteria offered the benefits of OLT to about 20% of our patients who would have otherwise been excluded from OLT under the more restrictive Milan criteria. The UCSF criteria also confer an advantage over the Pittsburgh criteria, which require information on microvascular invasion that is difficult to ascertain preoperatively without the attendant risks of biopsy [47].

Candidacy is now decided based on size and number of lesions (Milan, UCSF criteria, etc.). However, there are other important predictors of recurrence and patient survival that have not been included in the current selection system such as AFP/AFPL3% levels, tumor differentiation, and microvascular invasion. AFP is the most widely tested biomarker in HCC [22]. Lens culinaris agglutinin-reactive AFP (AFP-L3) is an isoform of AFP that is very specific for screening and diagnosis of HCC in the background of cirrhosis or hepatitis. However, the clinical usefulness of AFP-L3 in HCC has been inconsistent. Unfortunately, there has been large heterogeneity on the results of multiple studies when assessing AFP-L3% and AFP in the same population [48].

The relationship between AFP levels and tumor burden has been determined in HCC patients. Our group reported that tumor size and AFP were strongly related to the presence of microvascular invasion (MVI) on the explanted liver. AFP in conjunction with other markers such as des-gamma-carboxyprothrombin and AFP-L3 has been associated with tumor burden as well [49]. Because of the relationship with tumor burden and outcomes, several centers are now using highly elevated levels of AFP to rule out patients for liver transplantation. In 2012, Duvoux et al. published that levels of AFP greater than 1000 U/dL are associated with high risk of recurrence and suggested that AFP should be incorporated to the Milan criteria in order to select patients for liver transplantation [50].

Based on UNOS data, the 3 most common indications for liver transplantation in the USA are hepatitis C, alcoholic liver disease, and hepatocellular carcinoma. Interestingly, in 2010, HCC is already the second most common indication for transplantation, second only to hepatitis C. A significant amount of patients with HCC have concomitant HCV infection which could compromise outcomes in this subgroup of patients.

Reinfection of the liver graft by HCV is constant, and the natural history of HCV recurrence is accelerated compared with nontransplanted patients when serum HCV RNA remains detectable at LT. Approximately 20% to 30% of patients will develop cirrhosis within 5 years after LT.

The best strategy to prevent recurrence of HCV is to eradicate HCV infection prior to LT. Treatment before the development of injury to the graft in the early phase is currently not recommended because studies have shown that it is difficult to initiate antiviral therapy with IFN during the postoperative period and that it has a poor efficacy with remarkable side effects such as bacterial infections, hematological toxicity, and rejections, which lead to dose reduction or discontinuation of treatment.

HCV therapy should be initiated in the presence of severe and rapid progression of fibrosis with a higher risk of graft loss, especially in the setting of cholestatic hepatitis. Current regimens included antiviral therapy with PEG-IFN/RBV, and several studies have shown that a sustained virological response is achieved in 8%–45%. Three different systematic reviews of PEG-IFN/RBV after LT showed that the SVR rate is around 30% [51–53]. The duration of therapy is usually 48 weeks, and therapy is influence by several factors which influences the prognosis before and during therapy such as viral genotype, donor age, baseline viral load, IL28B donor and recipient, absence of prior antiviral therapy, severity of baseline fibrosis, adherence to therapy, duration of therapy, rapid virological response, and early virological response.

Due to the development of new drugs for the treatment of HCV infection, most experts believe that treatment of HCV recurrence after LT will change in the next few years. The role of triple therapy using PEG-IFN/RBV plus protease inhibitors is not clear. Verna EC et al. recently published the results of a multicenter study using triple therapy with Telaprevir in HCV recurrence after LT reporting increased sustained viral response rates exceeding those with standard treatment with PEG-IFN/RBV alone [54]. These results must be balanced with high rates of adverse events including increased risk of readmissions, kidney dysfunction, and death. There are now other protocols under investigation in patients with cirrhosis including noninterferon regimens. The role of these treatment combinations in the LT setting is still to be determined.

The timing and adequacy of HCC treatment before liver transplantation to control the disease are unclear. New minimally invasive strategies are being implemented to decrease tumor progression in patients with diagnosis of HCC while waiting to be transplanted.

The appropriate treatment alternative that should be used as neoadjuvant therapy prior to LT such as liver resection, radiofrequency ablation (RFA), transarterial chemoembolization (TACE), or combination remains under debate.

Considering the strength and existing evidence, it is recommended to treat patients waiting for transplant with local ablation and/or chemoembolization when waiting time is estimated to exceed 6 months. Furthermore, several cohort studies and a preliminary analysis of large registries suggest that bridging strategies with locoregional therapy are likely to be beneficial for patients waiting for 6 months or longer. The recommendation of bridging therapy is more important in UNOS T2 HCC patients to decrease dropout rates and achieve good posttransplant outcomes [55, 56]. A Markov-based cost-effectiveness analysis by Llovet et al. pointed out the benefits of neoadjuvant therapies when waiting time exceeded 6 months [5]. In our center, the decision to use bridging therapy is done on an individual basis by a multidisciplinary team integrated by hepatologist, radiologist, interventional radiologists, oncologist, radiation oncologists, and transplant surgeons. We usually recommend TACE and/or RFA in patients with UNOS stage 2 HCC prior to LT if 6 months or longer waiting time is expected in rapidly growing tumors or in large lesions close to the upper size limits of the Milan criteria.

Based on the International Consensus Conference held in December 2010 in Zurich, Switzerland, specific recommendations regarding downstaging cannot be properly made due to the lack of evidence, and further research is needed [22].

Although adjuvant therapy is currently used in most centers in the USA and Europe, further investigation is needed to determine the efficacy and timing of neoadjuvant treatment modalities for HCC in patients awaiting liver transplantation.

## Figures and Tables

**Table 1:** Patient demographics.

Patient demographics		
Age	56	(36–72)
Sex		
Male	78	81.30%
Female	18	18.70%
Incidental	31	32.30%
ALD	40	41.70%
HCV	48	50%
BT	19	29.20%
Size of greater lesion (mean)	2.3	(0.7–7.6 cm)
Single	51	54.40%
Vascular invasion	22	22.90%

Alcoholic liver disease (ALD);  hepatitis C cirrhosis (HCV);  bridging therapy (BT).

**Table 2:** Comparison of patients, characteristics.

Comparison of patients' characteristics Bridging therapy versus nonbridging therapy			
	BT	Non-BT	Sig
Age	59.4	55.9	0.47
Sex (male)	16	58	0.46
AFP	168	160	0.98
MELD	13	16	0.86
Waiting time	59.5	152.1	0.09
Vascular invasion	5	15	0.45
Multiple	7	33	0.32
Size > 3 cm	7	11	0.04*
HCV	11	36	0.26

*Statistically significant (*P* < 0.05).

Bridging therapy (BT);  alpha-fetoprotein (AFP);  hepatitis C cirrhosis (HCV).
